# Optimizing design parameters of 3D‐printed poly‐4‐hydroxybutyrate nipple scaffolds for nipple reconstruction

**DOI:** 10.1002/btm2.70010

**Published:** 2025-04-07

**Authors:** Xue Dong, Sophia Salingaros, Timothy Butler, Skander Limem, Jason A. Spector

**Affiliations:** ^1^ Laboratory of Bioregenerative Medicine & Surgery, Department of Surgery, Division of Plastic Surgery Weill Cornell Medical College New York New York USA; ^2^ Becton, Dickinson and Company (BD) Surgery Division Lexington Massachusetts USA; ^3^ Nancy E. and Peter C. Meinig School of Biomedical Engineering Cornell University Ithaca New York USA

**Keywords:** 3D printing, absorbable, mesh, nipple reconstruction, P4HB, scaffold

## Abstract

Nipple reconstruction in patients who undergo total mastectomy or nipple‐sparing mastectomy is currently limited by a consistent and significant loss of nipple projection over time, which can negatively affect patient satisfaction and quality of life. To address this issue, we have previously shown that 3D‐printed poly‐4‐hydroxybutyrate (P4HB) nipple‐shaped scaffolds promote long‐term maintenance of nipple projection in a rat model. Herein, we further optimize the 3D printing parameters (filament diameter and infill density) of absorbable P4HB latticework scaffolds as well as scaffolds fabricated from rolled P4HB knitted mesh to facilitate tissue formation with similar biomechanical properties of the native nipple, while maintaining long‐term shape and projection. Over 12 months of in vivo implantation in a dorsal, bilateral CV‐flap rat model of nipple reconstruction, 3D‐printed P4HB latticework and knitted mesh scaffolded groups demonstrated significantly greater maintenance in projection (80–100% of initial value) when compared to the Cook Biodesign® Nipple Cylinder (~40% of initial projection), resulting from the infiltration of healthy fibrovascular adipose tissue, which demonstrated biomechanical qualities that approached those of the native human nipple. Overall, our results demonstrate that using a 3D‐printed P4HB latticework and rolled P4HB knitted mesh scaffolds significantly improved long‐term results in our animal model of nipple reconstruction and hold promise for improving nipple reconstruction outcomes in future clinical practice.

**Translational Impact Statement**

We have engineered absorbable nipple scaffolds using a 3D‐printed P4HB latticework and rolled P4HB knitted mesh that demonstrated consistent maintenance of nipple projection and diameter over 12 months in a rodent model. As the P4HB scaffold degrades, it is replaced by fibrovascular adipose tissue, which approximates the proper shape, volume, and biomechanical qualities of the human nipple. These data demonstrate that such implants hold promise for clinical translation to provide long‐lasting projection for patients requiring nipple reconstruction after mastectomy.

## 1 INTRODUCTION

Breast cancer is the second most common malignancy diagnosed in women in the United States.^1^ Mastectomy, which involves removal of the breast tissue and nipple‐areolar complex (NAC), remains a common treatment modality (nearly 33.8% of diagnosed breast cancer patients), having been performed in approximately 110,000 patients in the United States in 2023.^1^, ^2^ Even as mastectomy techniques that spare the NAC (“nipple‐sparing mastectomy—NSM”) grow in popularity for appropriate cases, even the “spared” nipple loses nearly one‐third of its projection after 1 year.^3^, ^4^ The presence of a reconstructed and appropriately projecting nipple has been established as a significant factor in improving patient satisfaction post‐mastectomy by enhancing a woman's body image, quality of life, and psychosocial and sexual wellbeing.^5^, ^6^, ^7^ However, nearly all current options for nipple reconstruction (e.g., local skin flaps, autologous skin and tissue grafts, and acellular tissue substitutes) demonstrate significant projection loss (~70%) of the reconstructed nipple over time.^8^, ^9^ Loss of nipple projection typically begins within the first 3–6 months after NAC reconstruction due to insufficient structural support and contractile scar forces on the neo‐nipples.^10^, ^11^, ^12^

Our group has previously used 3D‐printed poly‐4‐hydroxybutyrate (P4HB) scaffolds filled with mechanically processed (minced or zested) autologous costal cartilage (CC) or an internal P4HB 3D latticework to engineer neo‐nipples with long‐term maintenance (1 year) of projection.^13^, ^14^ P4HB, a biopolymer used in a variety of FDA‐cleared, commercially available medical devices, was selected for its superior biocompatibility, tunable strength, and adjustable absorption rate.^15^, ^16^ Using particulate CC within the 3D‐printed P4HB scaffold as an infill support helped maintain nipple shape and projection and reduced the known risk of postoperative flap necrosis from rigid cartilage grafts.^17^ In addition, tissue infiltration between the cartilage particles allowed for the development of a fibrovascular cartilaginous tissue that resisted the contractile forces of the surrounding skin flap as the P4HB shell degraded. Compared to the preservation of ~100% of initial nipple projection in the CC‐filled groups after 12 months in vivo, the 3D‐printed P4HB scaffolds supported by an interior P4HB latticework, with 20% infill density and an external shell printed with 0.2 mm diameter printing filament, encountered a 25% loss of initial projection. It was hypothesized that this occurred due to rapid tissue infiltration and polymer degradation, leading to the loss of scaffold integrity at the “waist” of the neo‐nipples, resulting in premature buckling and occasional asymmetric nipple projection.^13^, ^16^, ^18^, ^19^

To optimize the clinical translatability (and simplicity) of this approach, we sought to produce engineered nipples with long‐term maintenance that did not require the addition of a cartilage particulate infill. Understanding the local tissue reaction and degradation rate of P4HB nipple scaffolds is crucial for the intended clinical application of this technology. Thus, in this study, we aimed to modify several parameters of the scaffold design, including filament diameter and infill density within the 3D‐printed latticework, thereby modulating scaffold degradation and tissue ingrowth rates to 1) improve implant‐based long‐term projection and 2) provide biomechanical properties similar to human nipples.

## 2 MATERIALS AND METHODS

### 2.1 Scaffold fabrication

**FIGURE 1:**
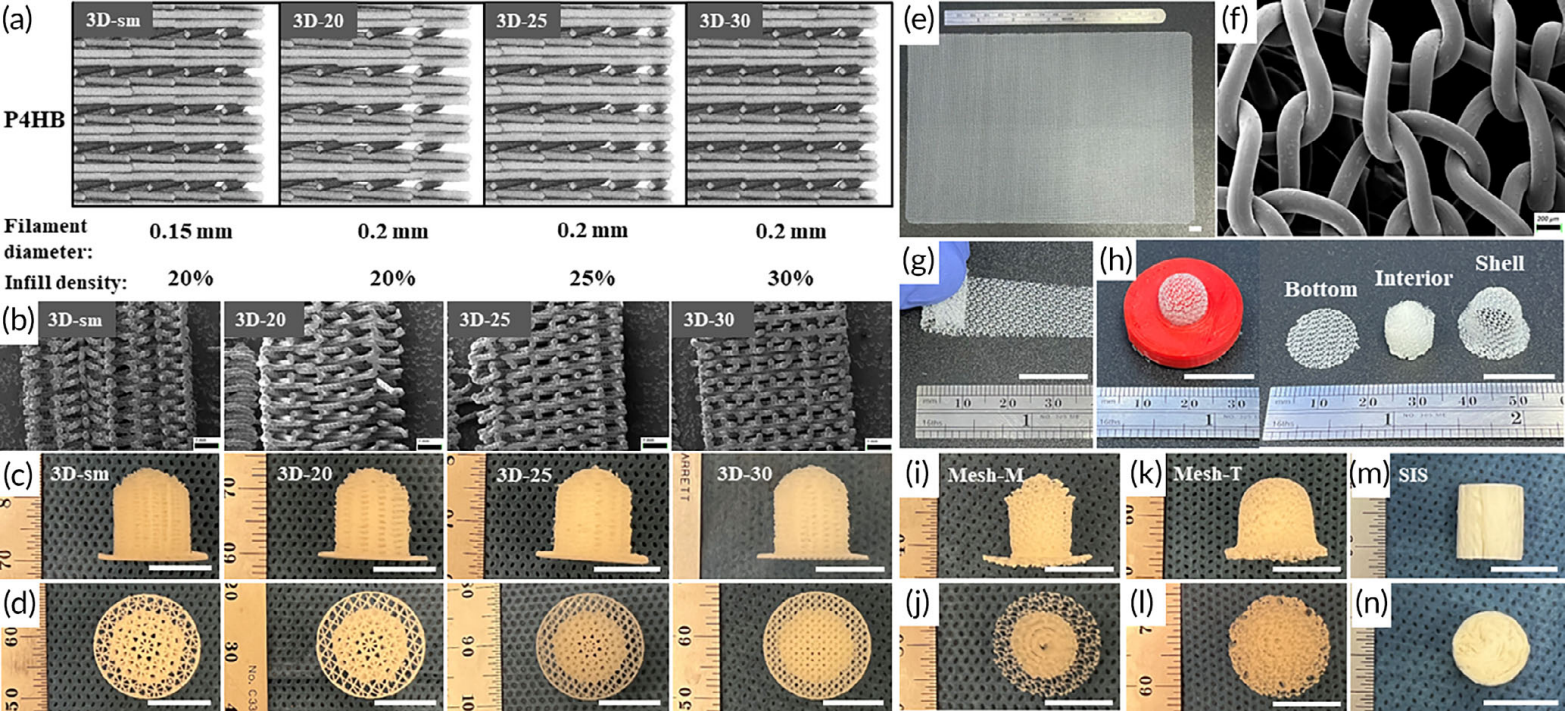
P4HB nipple scaffold design and fabrication. (a) Renderings of 3D‐printed latticework design with different parameters. (b) Representative SEM images of the cross‐section view of 3D‐printed P4HB nipple scaffolds (Scale bar = 1 mm). (c, d) Representative gross images of 3D‐printed P4HB nipple scaffolds: Side view, bottom view of the scaffold with flange extension. (e) Gross view of Phasix™ knitted mesh for fabricating scaffolds and (f) SEM of Phasix™ knitted mesh (Scale bar = 200 um). (g) Manually rolling mesh strip (1 cm wide) into a cylindrical shape (4–5 layers of mesh and 1 cm in diameter). (h) Mechanically thermoforming the dome‐shaped external shell with Phasix™ knitted mesh, and assembling the bottom, interior and shell into nipple scaffolds. (I, J) Representative gross images of manually rolled mesh scaffold (Mesh‐M): Side view, bottom view of the scaffold. (k, l) Representative gross images of mechanically thermoformed mesh scaffolds (Mesh‐T): Side view, bottom view of the scaffold. (m, n) Representative gross images of COOK Biodesign® Nipple Cylinder: Side view, bottom view of the scaffold. Scale bar = 200um in F and 1 mm in B. Scale bar = 1 cm in gross nipple images (c, d, e, g‐n).

Nipple scaffolds were designed using CAD software and printed using an Arburg 750‐3X Freeformer 3D printer (Lossburg, Germany) with chamber cooling to form a 1.0 × 1.0 cm (diameter × height, equivalent to the control group Cook Biodesign® Nipple Cylinder) dome‐topped cylinder with different P4HB filament diameters (0.15 mm or 0.2 mm) and infill densities (20%, 25% or 30%): **3D‐sm** (0.15 mm, 20%), **3D‐20** (0.2 mm, 20%), **3D‐25** (0.2 mm, 25%) and **3D‐30** (0.2 mm, 30%) (Figure 1a–d). The P4HB polymer was manufactured and provided by BD (Becton, Dickinson & Company, Lexington, MA). Briefly, nipple scaffolds were 3D‐printed using P4HB pellets within a nozzle temperature range of 190°C to 220°C and a chamber temperature cooled down between 12°C and 16°C to speed up P4HB crystallization, minimize warping, and enhance print quality and structural integrity. The three‐dimensional latticework was designed to provide structural support and increase the surface area between the scaffold and the host tissue. In comparison to prior designs, in this study, greater infill densities of 25% and 30% were designed to address the previously observed premature scaffold buckling. To encourage more rapid tissue infiltration and timely scaffold degradation, we designed 3D‐printed nipple scaffolds without an external shell, which was shown in previous studies to impede infiltration. Additionally, to better understand how thinner filaments would affect the same variables, we also fabricated 3D scaffolds with a 0.15 mm filament diameter. A 2.5 mm wide flange along the base of the scaffold was designed and printed with the same diameter P4HB filament as the body of the scaffold, to provide stability and prevent tipping or flipping after initial implantation (Figure 1c,d). Manually rolled P4HB knitted mesh scaffolds (**Mesh‐M**) (Figure 1e–g,i,j) and mechanically thermoformed P4HB knitted mesh scaffolds (**Mesh‐T**) (Figure 1h,k,l) of the same size (1 × 1 cm, 4 to 5 layers of rolled mesh) were fabricated from commercially available Phasix™ Mesh (BD, Franklin Lakes, NJ), a knitted P4HB monofilament mesh, for comparison. It should be noted that Phasix™ mesh is currently indicated for use in the reinforcement of soft tissue repair, where weakness exists, and is not indicated for use in nipple reconstruction. The mechanically thermoformed mesh scaffolds contained a bottom, rolled interior, and external shell that was thermally fused together (Figure 1h). The Cook Biodesign® Nipple Cylinder (reference part number: C‐NRC‐1.0X, 1.0, 1.0 cm in diameter × height) was included as a control **SIS** group (Figure 1m,n). This cylinder is a rapidly absorbable extracellular collagen matrix derived from porcine small intestinal submucosa (SIS) and is the only US FDA‐authorized device that is indicated for use as a nipple reconstruction scaffold. All P4HB scaffolds were sterilized using ethylene oxide prior to in vivo study. Details of each study group are summarized in Table 1.

**TABLE 1:** Summary table of study groups.

Group	3D‐sm	3D‐20	3D‐25	3D‐30	Mesh‐M	Mesh‐T	SIS
Infill density (%)	20	20	25	30	N/A	N/A	N/A
Filament diameter (mm)	0.15	0.20	0.20	0.20	0.17	0.17	N/A
Flange	+	+	+	+	+	+	−
3D‐printed	+	+	+	+	−	−	−
Fabrication material	P4HB filament	P4HB filament	P4HB filament	P4HB filament	Knitted P4HB Mesh	Knitted P4HB Mesh	Porcine small intestinal submucosa

### 2.2 Animal model

All animal care and experimental procedures followed the Guide for the Care and Use of Laboratory Animals and were approved by the Weill Cornell Medicine Institutional Animal Care and Use Committee (protocol # 2011–0036). A total of 58 male Sprague Dawley rats (two extra rats were used to replace the four nipples that had complications), with a weight ranging from 250 to 300 g, were included in the study. Each animal was assigned a unique number, and a computer program randomly selected animals to different treatment groups. For nipple reconstruction, a CV‐flap incision pattern was designed using silicone sheet stencils with measurements matching the dimensions of 3D‐printed scaffolds, as previously described.^13^, ^14^ After the animals were anesthetized and prepped, CV flaps consisting of full‐thickness skin were raised bilaterally on the dorsum. The two arms of the CV flap were approximated to form a cylindrical pocket, into which a scaffold was placed. Incisions were closed with interrupted 4–0 nylon sutures, and the reconstructed nipples (two identical scaffolds per rat) were dressed with a protective hollow plastic cylinder for 10 days post‐operatively. Animals were euthanized after 1, 3, 6, and 12 months followed by nipple explantation for further analysis (*n* = 4 nipples per group/time point, total of 112).

### 2.3 Contour analyses

In addition to the qualitative assessment of the size, shape, and structural integrity of nipples, the contour and projection were assessed quantitatively in a standardized fashion using calipers from both the coronal and sagittal perspectives prior to explantation and compared to baseline measurements taken at the time of implantation, as previously described.^13^, ^14^ Projection was defined as the distance from the base to the tip of the nipple at the midline axis with no exogenic compression applied during measurement. Similarly, the mean diameter was calculated by averaging the nipple diameters measured 1) along the long axis of the rats (sagittal) and 2) the axis perpendicular to the long axis (coronal) from the overhead perspective.

### 2.4 Volumetric analyses

At explantation, each neo‐nipple construct was carefully dissected from the skin and surrounding tissue. The volume of each construct was assessed immediately after explantation using the Inveon Pre‐clinical MicroPET/CT/SPECT (CTI/Siemens, Knoxville, TN). Regions of Interest (ROI's) were then created in digitally reconstructed images by selecting voxels using thresholds for pixel intensity. A combined 3D ROI created from the coronal, axial, and sagittal views was generated to acquire a precise volume measurement of the scaffold and tissue formation at each time point.

### 2.5 Assessment of tissue ingrowth

Observation of neo‐nipple constructs was conducted at an explanation to grossly evaluate the tissue ingrowth, capsule formation, and vascularization. Each explanted nipple was divided in half along the construct's long axis with one‐half snap frozen in liquid nitrogen and subsequently stored at −80°C for biomechanical testing and the other half prepared for histological evaluation: each sample was fixed in 10% formalin, processed with the Tissue‐Tek VIP 6 Vacuum Infiltration Processor, paraffin‐embedded and sectioned at 15 μm thickness. Slides were stained with hematoxylin & eosin (H&E), immunofluorescent (IF) stained for CD31 (endothelial cell) (Biolegend, San Diego, CA), CD80 (M1‐like macrophage) (Thermo Fisher Scientific, US) and CD206 (M2‐like macrophage) (ABCAM, Cambridge, UK) to assess the condition of overall tissue ingrowth, neovascularization, inflammation and macrophage phenotype within the scaffolds. Sections were also stained with Masson's Trichrome to assess the newly formed extracellular collagen matrix. Quantification of CD31, CD80, CD206, and collagen expression was performed using Image J (U.S. National Institutes of Health, Bethesda, MD). Serial sections of explanted neo‐nipples were made, and expression was quantified by the percentage area of positive staining from three separate sections of each cut sample with five randomly chosen high‐powered fields from each section (100X magnification for Masson's Trichrome, 200X for IF). Tissue ingrowth within the nipple constructs was evaluated by a blinded board‐certified veterinary pathologist.

### 2.6 Biomechanical analyses

Unconfined compression testing was performed on defrosted neo‐nipples by dividing them in half (sectioned apex to base) and placing them horizontally with the sectioned side lying flat, as previously described.^13^, ^14^ In this configuration, specimen height was approximately 5 mm. Each sample was subjected to compression testing using a mechanical tensile tester (QTestTM/1 L, MTS, USA) equipped with two opposing flat plates. To remove potential slack from the specimen setup, a 0.1 to 0.9 N pre‐load was applied to each specimen without compressing the specimen height. Specimens were then compressed 1 mm (20% strain) from their original height. Ten compression cycles were performed at 20 mm/min to evaluate the stiffness of each construct and the extent of elastic recovery. Compressive modulus was calculated using data from the second load cycle as the slope between 0.2 mm and 1 mm displacement to eliminate the artifact caused by a take‐up of slack and seating of the specimens. The recovery of the nipple constructs under cyclic forces was also analyzed. One group of nipple constructs was tested prior to implantation (time 0, T0) while a separate group was implanted and then tested after each explantation time point (*n* = 4 nipples per group).

### 2.7 Molecular weight testing

The weight‐average molecular weight (Mw) of pre‐implanted and digested explanted P4HB scaffolds was determined with a TOSOH EcoSEC HLC‐8320 gel permeation chromatography (GPC) system with a refractive index (RI) detector at 35°C using a PLgel MIXED‐C column (Agilent Technologies, Inc.) and chloroform (CHCl_3_) eluent (1.0 mL/min). Samples were dissolved in chloroform at 1 mg/mL and injected using 95 μL. The calibration curves for GPC analysis were obtained using polystyrene standards with a narrow molecular weight distribution.

### 2.8 Scanning electron microscopy

Explanted nipples were digested with collagenase to remove fibrotic tissue, and scaffold samples were sputter coated with a gold/palladium coating for a total of 90 seconds with stage rotation (3 × 30 seconds to avoid heating samples) at the manufacturer's recommended settings, using a Ted Pella Cressington Auto 108 sputter coater. Samples were imaged with a Zeiss EVO25 SEM with a Secondary Electron Detector, accelerating voltage 5 kV, under high vacuum (<1 Pa).

### 2.9 Statistical analyses

The data were summarized in the form of absolute values or percentages as means ± standard deviation. To compare preservation of projection, diameter, and volume between the groups, one‐way analysis of variance (ANOVA) was performed. One‐way ANOVA was also performed to analyze expression levels of CD80, CD206, CD31, and collagen deposition between groups at all time points. Tukey's multiple comparisons test was used post‐hoc to identify the difference between each group. The level of statistical significance for hypothesis testing was set at α = 0.05. All statistical analyses were performed using Prism 8.0 (GraphPad Software, Inc., La Jolla, CA).

## 3 RESULTS

### 3.1 Appearance of neo‐nipples

**FIGURE 2:**
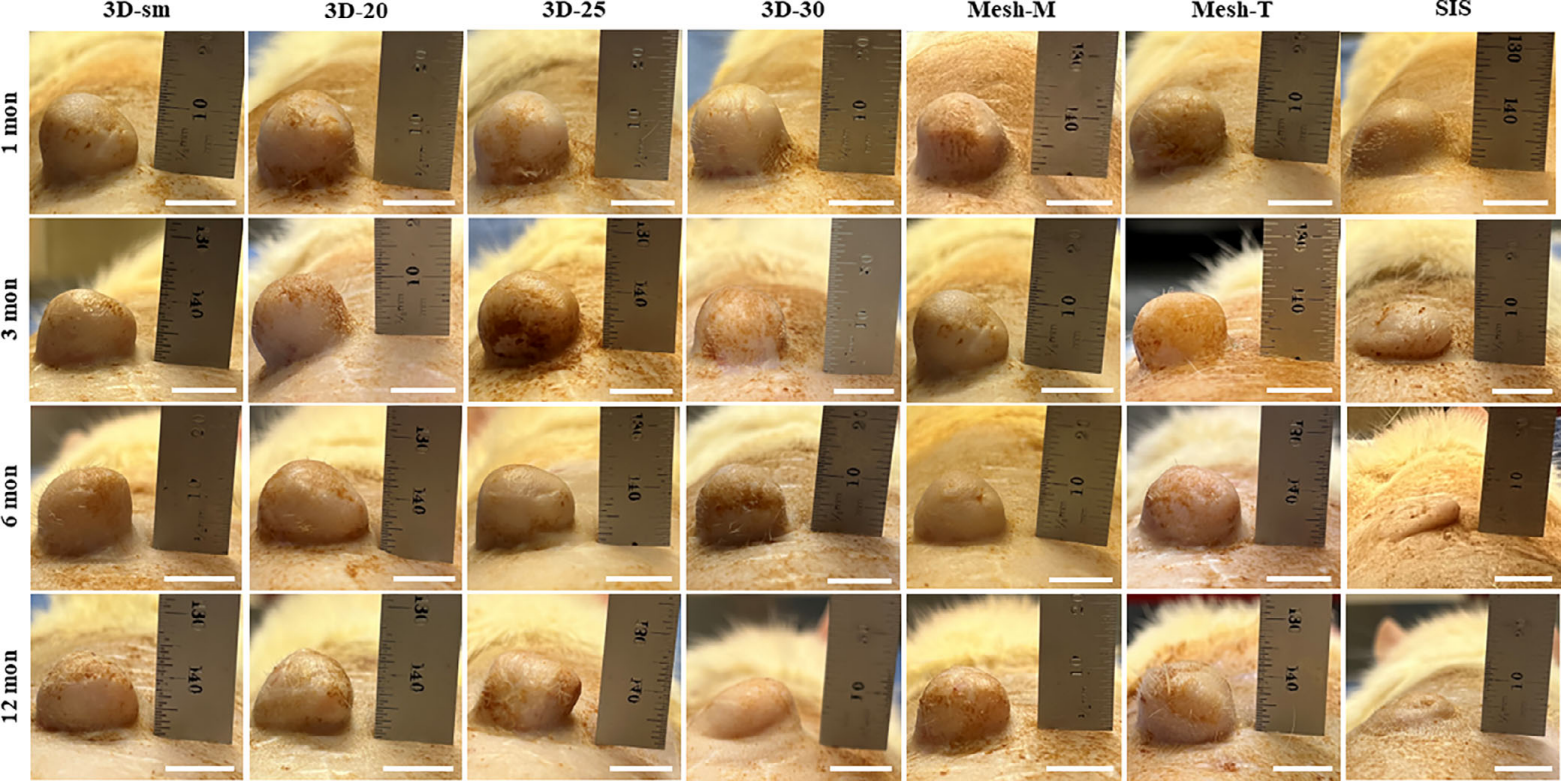
Representative gross images of neo‐nipples after 1, 3, 6, and 12 months. Compared to reconstructed nipples using either 3D‐printed or rolled mesh P4HB, the SIS group lost a significant amount of projection. Scale bar = 1 cm in gross nipple images.

Upon close gross examination, nearly all reconstructed nipples healed normally with appropriate contour, position, and color (Figure 2), with the exception of four nipples that were lost due to exposure (secondary to animal biting) and one nipple lost due to immediate postoperative flap necrosis. The lost nipples were replaced. There were no other perioperative adverse events, implant dislodgements, or surgical site infections related to the implanted nipple scaffolds.

#### 3.1.1 Preservation of neo‐nipple diameter and projection

**FIGURE 3:**
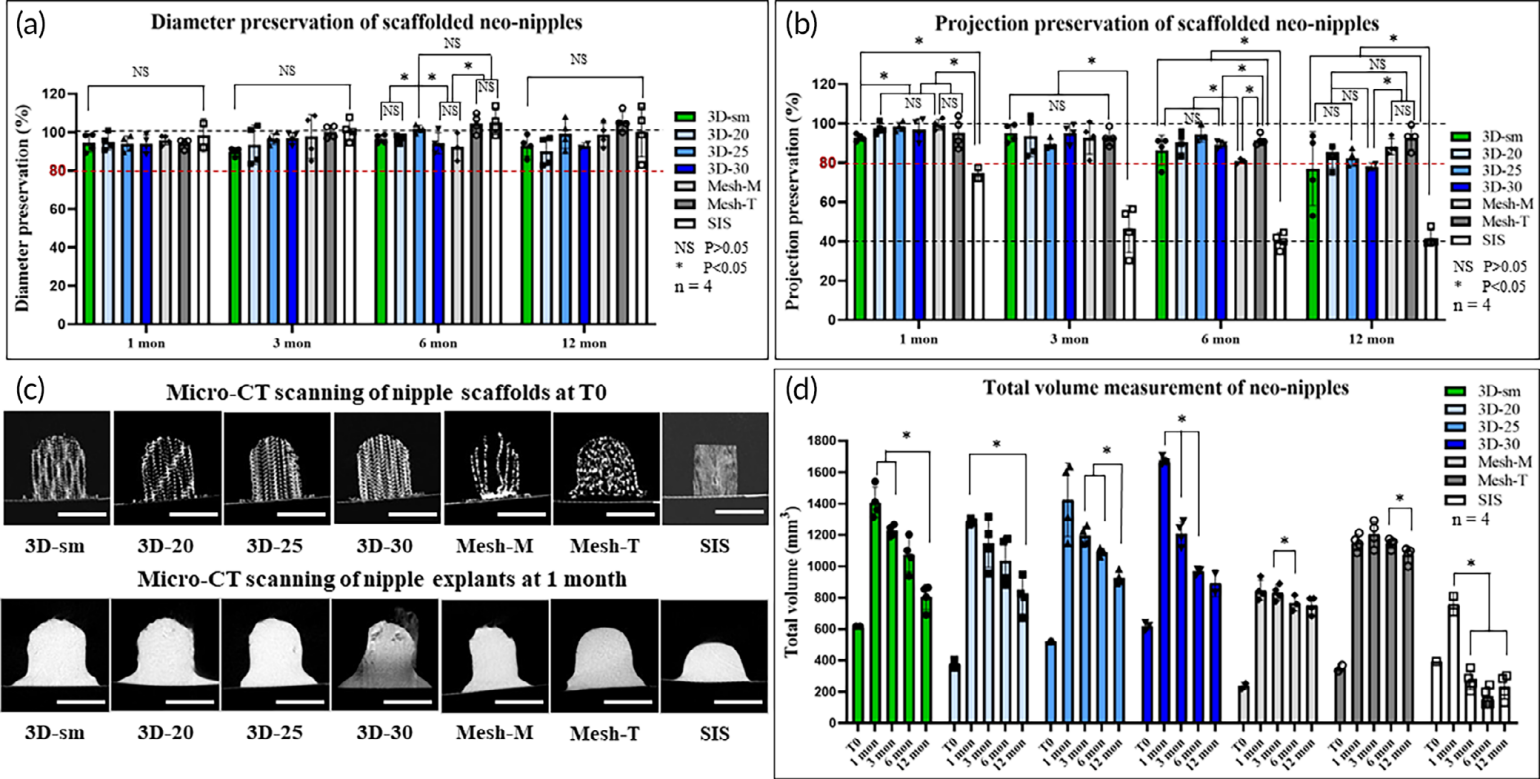
Dimensional and volumetric assessment of neo‐nipples after 1, 3, 6, and 12 months. (a) Diameter preservation of neo‐nipples; there were no significant differences between groups. (b) Projection preservation of neo‐nipples. (c) Representative CT‐scanned images of nipple scaffolds at pre‐implantation (T0, top panel) and after 1 month in vivo (lower panel). Nearly 100% of the interior space was filled by host tissue infiltration as early as 1 month after implantation. (d) Total volume of nipple scaffolds prior to implantation (scaffold‐only, T0) and neo‐nipples (scaffold + tissue ingrowth) after implantation. * Indicates a significant difference between the groups, *p* < 0.05. NS indicates no significant difference between the groups, *p* > 0.05. *n* = 4 nipples per group/time point. Scale bar = 1 cm.

The diameter of all scaffold groups was well maintained over time with achieving nearly 100% preservation after 12 months (3D‐sm: 93.0 ± 5.5%, 3D‐20: 90.0 ± 7.5%, 3D‐25: 99.2 ± 7.6%, 3D‐30: 93.0 ± 1.5%, Mesh‐M: 98.8 ± 6.5%, Mesh‐T: 105.5 ± 5.3%, SIS: 100.3 ± 13.1%, *p* > 0.05) (Figures 2 and 3a). There were no significant differences in diameter observed between different 3D‐printed P4HB scaffolds, manually rolled or mechanically thermoformed mesh scaffolds, or between P4HB scaffolds and the SIS device after 12 months. Only the base of neo‐nipples remained in the SIS group for diameter measurement after 12 months (Figure 2).

The projection of neo‐nipples with 3D‐printed P4HB scaffolds slightly decreased over time, achieving ~80% projection preservation after 12 months (3D‐sm: 77.0 ± 18.8%, 3D‐20: 82.7 ± 5.5%, 3D‐25: 82.4 ± 4.1%, 3D‐30: 78.0 ± 1.9%) (Figures 2 and 3b). In comparison, the projection of manually rolled and mechanically thermoformed mesh groups was well maintained across all time points (Mesh‐M: 99.3 ± 2.7% at 1 month, 92.7 ± 8.3% at 3 months, 80.8 ± 1.3% at 6 months and 88.3 ± 4.0% at 12 months; Mesh‐T: 95.3 ± 7.6% at 1 month, 93.0 ± 4.2% at 3 months, 91.6 ± 2.7% at 6 months and 92.7 ± 5.9% at 12 months). The SIS device lost significant projection by 1 month (74.8 ± 3.9%) and retained only 40% of the initial projection at later time points (46.4 ± 11.9% at 3 months, 40.3 ± 4.1% at 6 months and 41.7 ± 4.1% at 12 months).

#### 3.1.2 Volumetric analysis

**FIGURE 4:**
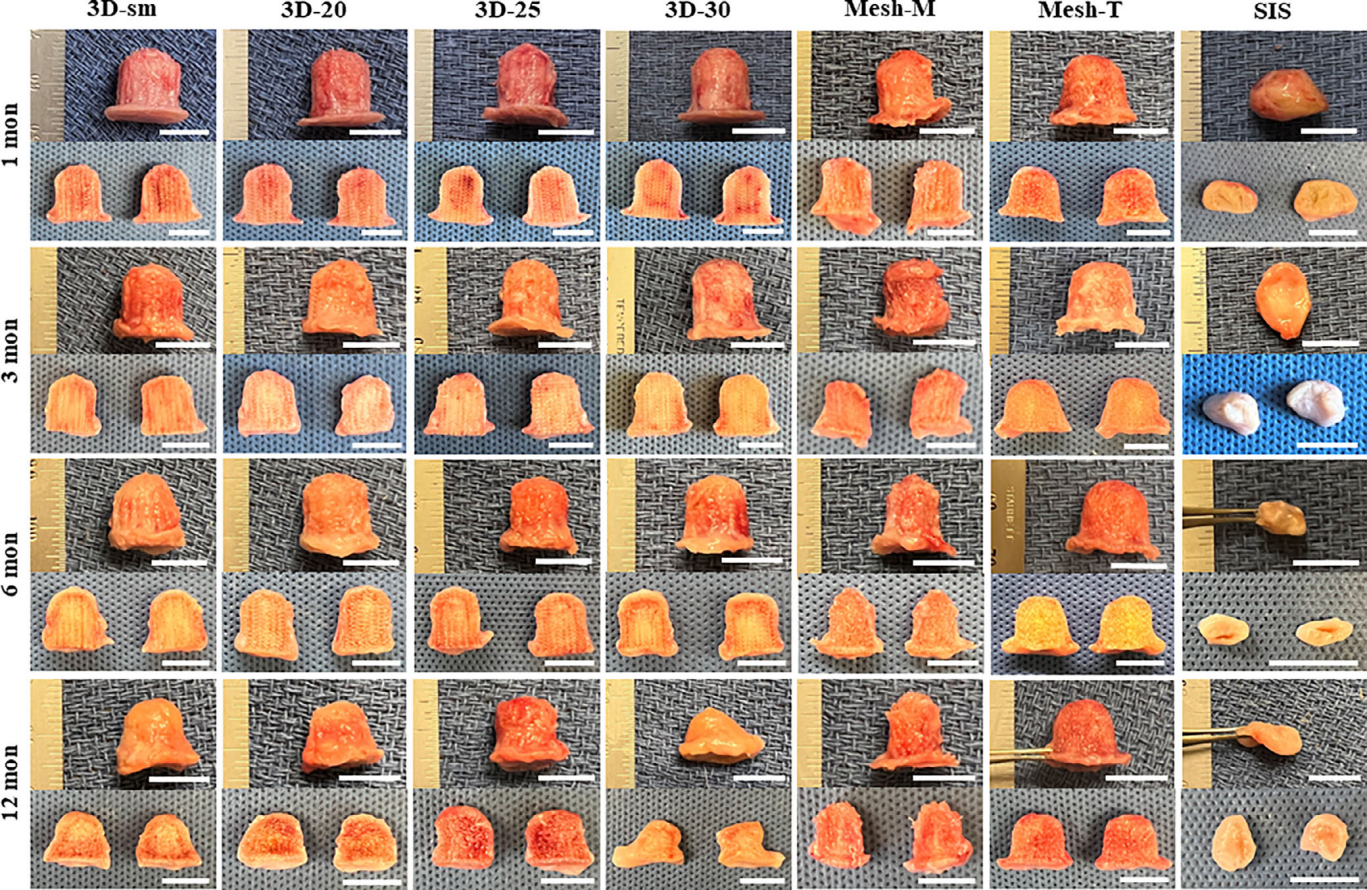
Representative gross images of neo‐nipple explants after 1, 3, 6, and 12 months from the side view of the whole explant (upper pane) and explant cross‐sections (lower pane). Vascularized tissue formation was evident in all neo‐nipple groups as early as 1 month after implantation, and degradation of scaffold material over time in 3D‐printed P4HB scaffolded groups and more rapid degradation in the SIS group was grossly apparent. Scale bar = 1 cm.

Volumetric measurement demonstrated a significant volume increase between pre‐implantation (T0) and 1 month in all P4HB groups due to rapid neotissue formation within the previously hollow lattice structure of the scaffolds (Figure 3c), with the 3D‐printed P4HB groups demonstrating near‐complete tissue infiltration as early as 1 month after implantation (Figures 3d and 4). A steady decrease in peak volume was noted in 3D‐printed P4HB groups, owing to the continuous degradation of P4HB, with the greatest volume decrease in 3D‐sm and 3D‐30 groups between 1 and 12 months (3D‐sm: 803.9 ± 80.0 mm^3^ at 12 months, a 42.9% decrease from 1‐month, 3D‐20: 811.8 ± 114.1 mm^3^ at 12 months, a 36.7% decrease from 1‐month, 3D‐25: 928.4 ± 39.8 mm^3^ at 12 months, a 34.8% decrease from 1‐month, 3D‐30: 892.6 ± 85.6 mm^3^ at 12 months, a 46.6% decrease from 1‐month). However, in all these groups, the construct volume at 12 months remained significantly higher than T_0_, because of tissue ingrowth and maturation. In contrast, the volume of manually rolled and mechanically thermoformed mesh groups demonstrated minimal change over 12 months (Mesh‐M: 752.2 ± 54.2 mm^3^ at 12 months, an 11.3% decrease from 1‐month; Mesh‐T: 1073.7 ± 49.8 mm^3^ at 12 months, a 6.9% decrease from 1‐month), remaining as intact nipple scaffolds with a well‐preserved flange observed grossly at explantation after 12 months, largely because of the minimal degradation of the P4HB mesh fibers. Similar to the loss of projection, a large reduction of total volume was observed in the SIS group as early as 3 months after implantation, primarily due to absorption of the implanted SIS material and contraction of the skin flaps (759.3 ± 71.1 mm^3^ at 1 month, 274.9 ± 61.4 mm^3^ at 3 months, 171.3 ± 61.1 mm^3^ at 6 months and 231.0 ± 78.7 mm^3^ at 12 months (a 69.6% decrease from 1‐month), respectively).

### 3.2 Assessment of tissue formed within neo‐nipples

#### 3.2.1 Histologic analysis of neo‐nipples

**FIGURE 5:**
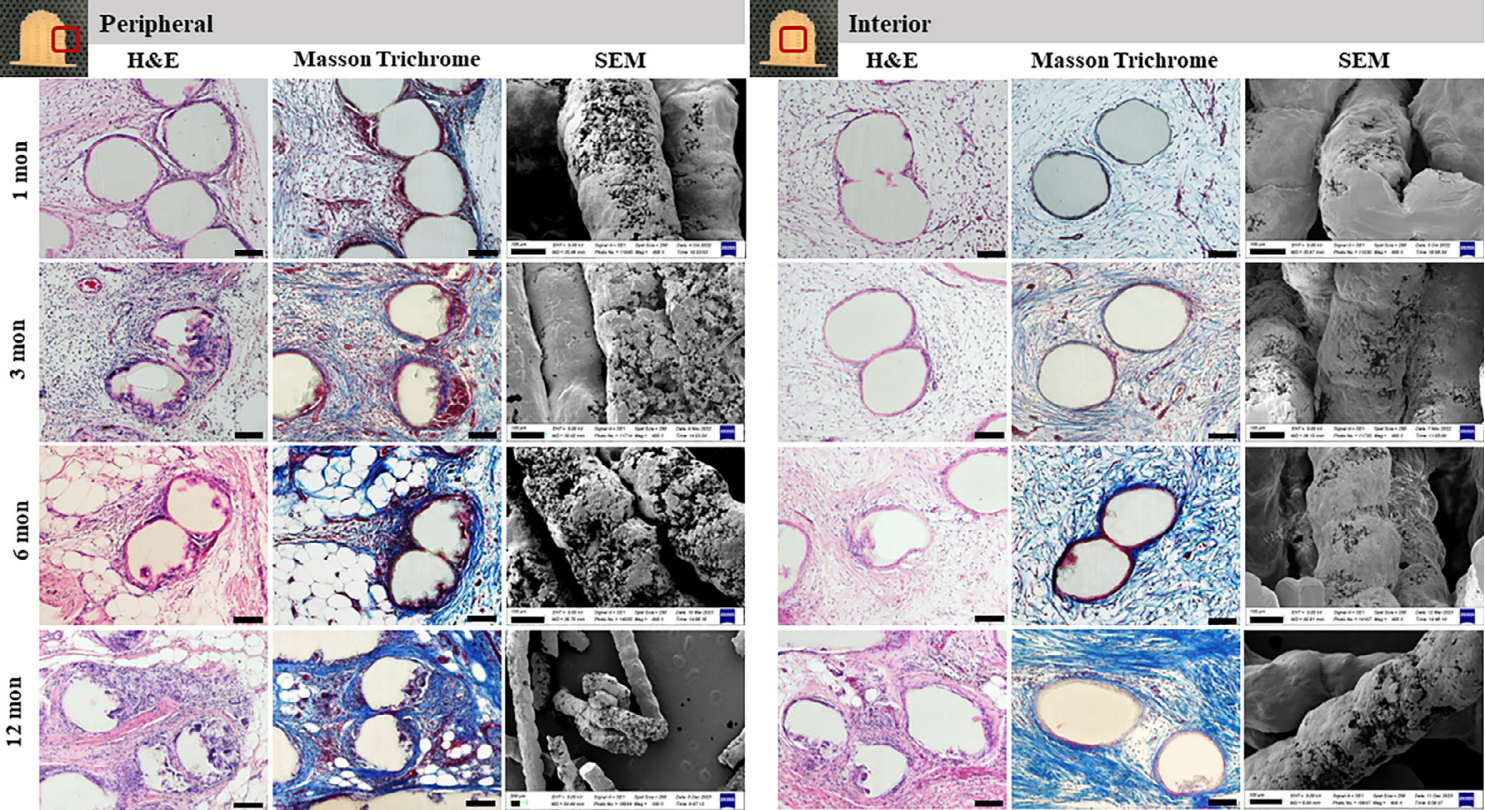
Tissue histologic assessment in 3D‐20 scaffolded neo‐nipples after in vivo implantation (left for peripheral, right for interior as indicated). H&E (first column) and Masson's Trichrome staining (second column) demonstrated overall cellular infiltration and collagen deposition in the nipple explants, accompanied by increased surface degradation of P4HB filaments over time seen on SEM images (third column). In addition to fibrovascular tissue, adipose tissue formation was seen within scaffolds, particularly along the periphery at 6 and 12 months. After 12 months, there was no remaining lattice structure on the surface of P4HB scaffolds with only scattered filaments remaining. Scale bar = 100 μm in H&E (first column) and Masson's Trichrome staining (second column). Scale bar = 100 μm in SEM images (third column).

**FIGURE 6:**
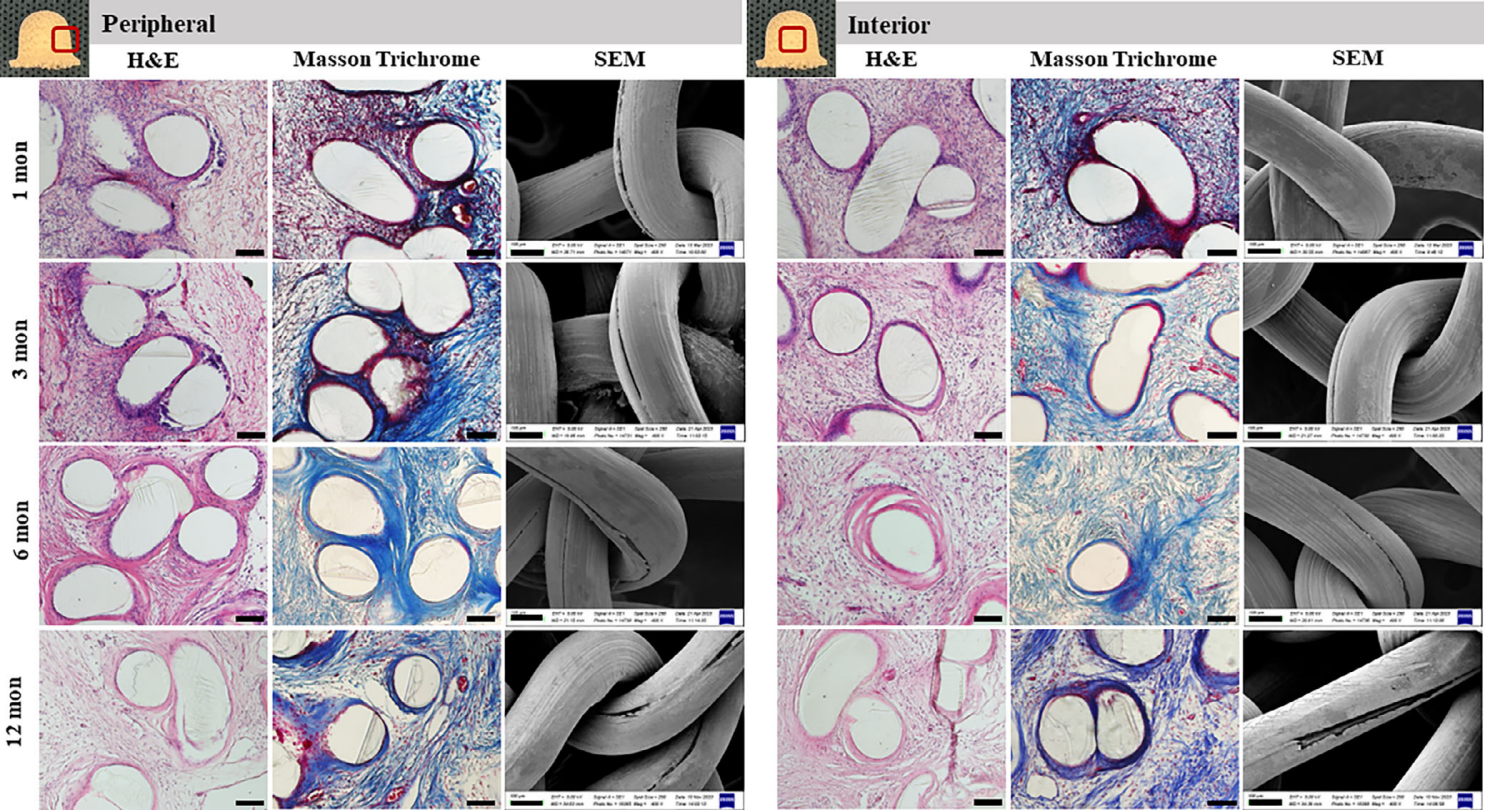
Tissue histologic assessment in Mesh‐T scaffolded neo‐nipples after in vivo implantation (left for peripheral, right for interior as indicated). H&E (first column) and Masson's Trichrome staining (second column) demonstrated overall cellular infiltration and collagen deposition. Minimal surface degradation of mesh fibers was noted over time (seen in SEM images, third column). Intact mesh fibers were gradually encapsulated by distinct layers of fibrous tissue at 6 and 12 months. Scale bar = 100 μm.

Upon explantation, all nipple constructs were carefully dissected from the skin and found to be invested by a very thin layer of filmy capsular material characterized by a smooth surface and a pink coloration (Figure 4). Histological analysis confirmed the microCT findings that all P4HB scaffolds were filled with vascularized tissue by 1 month. Robust inflammatory cell infiltration after 1 month was noticed in all scaffolds as an expected response to foreign material as well as surgical trauma. Increased fibrovascular tissue, abundant extracellular matrix deposition between P4HB filaments, and reduction of the inflammatory infiltrate were evidenced over 12 months in vivo (Figures 5 and 6, Supplementary Figures [Link], [Link]). Masson's Trichrome staining revealed early collagen matrix deposition with a disorganized thin fiber network throughout the scaffold by 1 month, which gradually increased and matured into a more aligned thick fiber network over 12 months. Collagen content in the scaffolds increased between 1 and 6 months but without statistical significance (Figure 8H). Collagen content in the 3D‐printed P4HB scaffolds remained consistent between 6 and 12 months. In addition to fibrovascular tissue, adipose tissue formation was seen within scaffolds after 6 months, particularly along the periphery.

Histopathologic examination of the 3D‐printed P4HB scaffolds at all timepoints revealed fibrovascular stromal proliferation within and around the scaffolds, with variable numbers of interstitial mononuclear cell infiltrates, including lymphocytes, plasma cells, and macrophages. Initial degradation of P4HB filaments was observed as early as 1 month in all 3D‐printed groups, especially along the construct periphery, as noted by pitting/erosion at the surface of the filaments from SEM. After 12 months, the greatest degradation of P4HB filaments was found along the periphery, with the outermost layer and flange portions fully disintegrated. Abundant multinucleated giant cells were observed in the vicinity of the degrading scaffold structure over 12 months. Compared to the periphery, the central portion of 3D‐printed P4HB scaffolds experienced moderate tissue infiltration with fewer inflammatory cell infiltrates and delayed P4HB filament degradation. Notably, some explants from 3D‐sm (smaller filament diameter, i.e., highest surface‐area‐to‐volume ratio) and 3D‐30 (highest infill density) groups encountered almost complete scaffold degradation with significant accumulation of inflammatory cells throughout the scaffold between 6 and 12 months, with few intact P4HB filaments present by 12 months (Supplementary Figures S1 and S3).

In comparison, minimal degradation of P4HB filaments occurred in both mesh groups (Mesh‐M and Mesh‐T) over 12 months, with grossly intact mesh fibers becoming gradually encapsulated by distinct layers of fibrous tissue at 6 and 12 months (Figure 6 and Supplementary Figure S4). Large amounts of variably loose and dense connective tissue with extracellular matrix deposition were present between mesh fibers at the early time points, which were maintained over time without significant differences (Figure 8h). Notably fewer mononuclear cells or giant cells were noticed across all time points in the mesh groups when compared to the 3D‐printed P4HB scaffolds.

Unlike all P4HB scaffolds, the compacted extracellular matrix of the SIS group was initially infiltrated by more mononuclear and polymorphonuclear inflammatory cells after implantation. This was followed by rapid absorption and replacement of the SIS material by host tissue (Supplementary Figure S5). The initial compacted and dense SIS collagen matrix demonstrated the highest collagen content at 1 month, which gradually decreased over time as the SIS cylinder was progressively absorbed with minimal replacement by host tissue (Figure 8h). The decrease was not statistically significant, but this transition was consistent with the rapid loss of projection and volume observed between 1 and 6 months.

#### 3.2.2 Immunofluorescent staining of neo‐nipples

**FIGURE 7:**
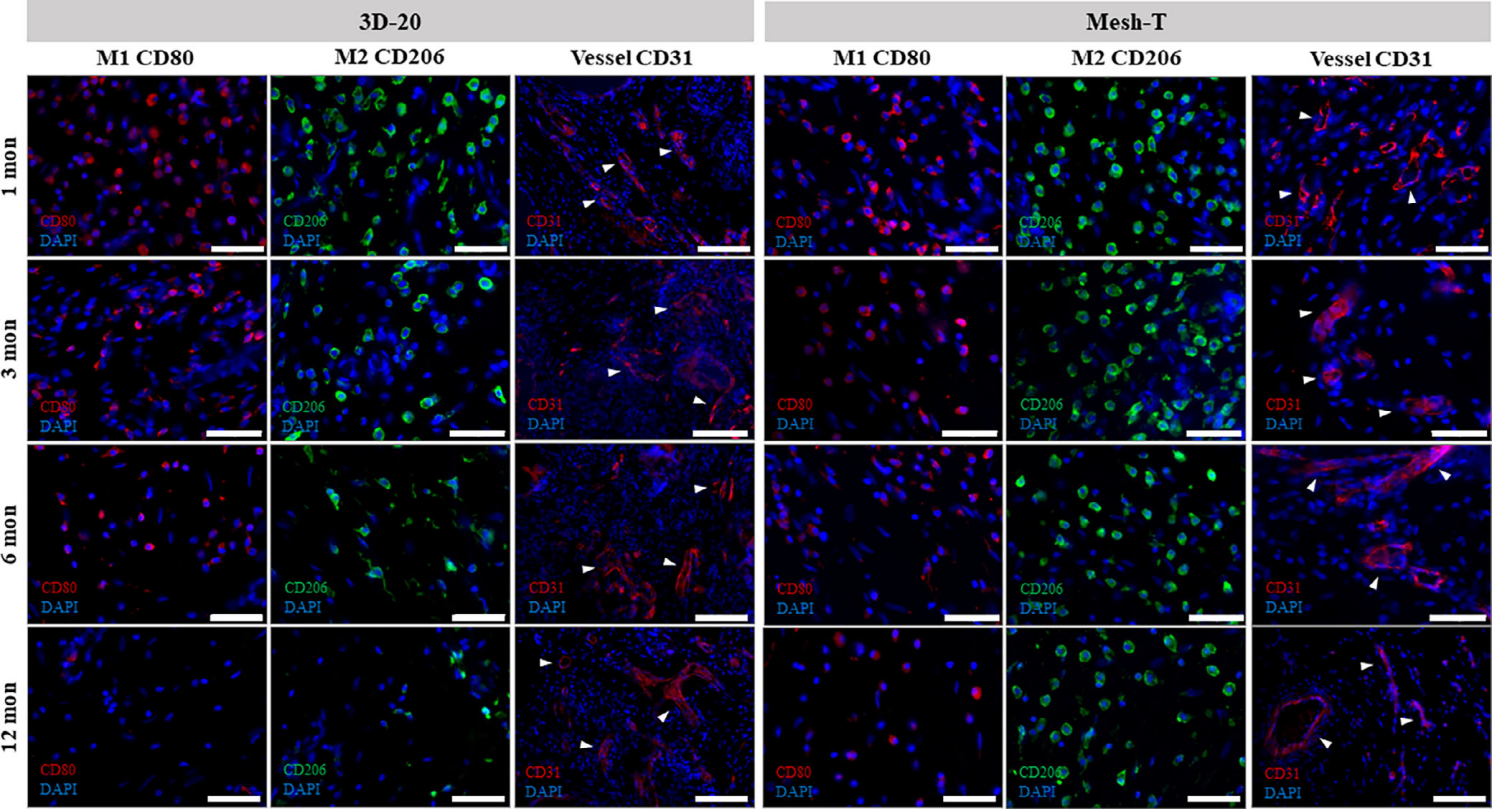
Identification of macrophage phenotypes and blood vessel formation in 3D‐20 and Mesh‐T scaffolded neo‐nipples after in vivo implantation (left for 3D‐20 group, right for Mesh‐T group). Representative images demonstrated the expression of CD80 (M1‐like macrophages in red, first column), CD206 (M2‐like macrophages in green, second column), CD31 (vessels in red, third column) and DAPI (blue). Note predominance of M2‐like macrophages and increased size of vessels (white arrow heads demonstrating luminal structures) over time. Scale bar = 50 μm.

**FIGURE 8:**
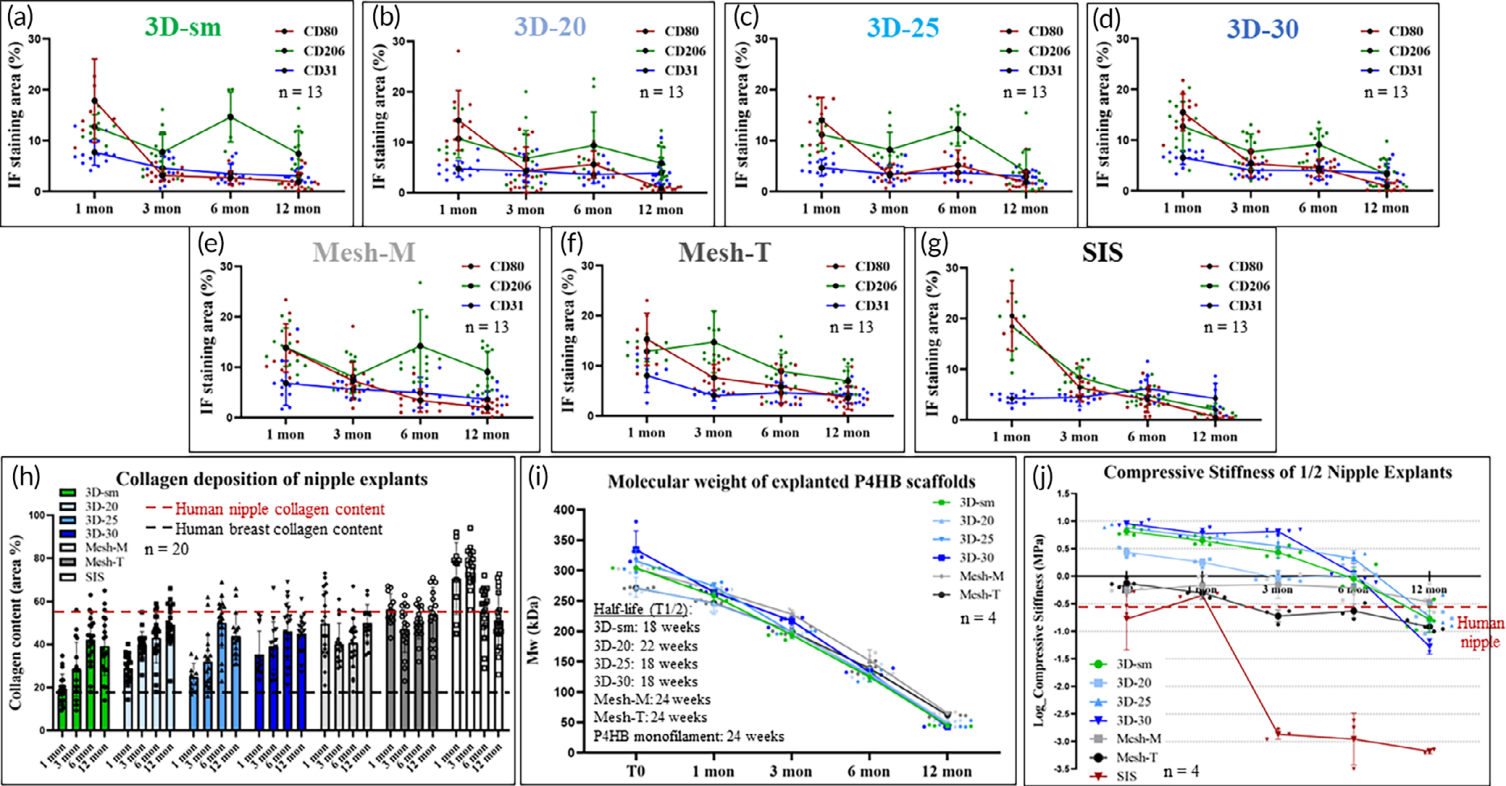
Quantitative assessment of macrophage phenotype, vascularization, collagen content, molecular weight, and stiffness of explanted neo‐nipples after in vivo implantation. (a–g) Quantification of CD80, CD206, and CD31 expression in explanted neo‐nipples as percent area (%) after 1, 3, 6, and 12 months (a: 3D‐sm, b: 3D‐20, c: 3D‐25, d: 3D‐30, e: Mesh‐M, f: Mesh‐T, g: SIS) (*n* = 13, means ± SD as shown). (h) Collagen content was assessed with Masson's Trichrome and measured by Image J as percent area (%) (*n* = 20, means ± SD as shown). (i) Molecular weight of all explanted scaffolds decreased over time (*n* = 4 nipples per group/time point). The half‐life (T1/2) of 3D‐printed P4HB groups after in vivo subcutaneous implantation decreased while mesh groups basically remained unchanged. (j) Compressive stiffness testing of neo‐nipples by sectioning a neo‐nipple in half (apex to base) and placing it horizontally with the sectioned side lying flat (presented in log scale) (*n* = 4 nipples per group/time point). Elastic modulus of the 3D‐printed P4HB scaffolds gradually decreased, approaching the previously reported human nipple reference stiffness (0.257 MPa), while both mesh groups decreased slightly but remained unchanged over 12 months within the range of 0.12 to 0.78 MPa.

Luminal endothelial cells within putative vascular structures, as evidenced by CD31 expression, were noted as early as 1 month in all P4HB groups, especially along the periphery; quantification of CD31 expression demonstrated extensive vessel formation (5–10% area stained) in all groups, especially the 3D‐sm, 3D‐30, Mesh‐M, and Mesh‐T groups at 1 month (Figure 8a–g). The intensity of CD31 staining slightly decreased after 1 month but remained mostly unchanged throughout 12 months in all 3D‐printed P4HB and mesh groups, with a more well‐developed vasculature seen after 6 months (Figure 7). The SIS group had a slight increase in vessel intensity over 6 months, similar to the other P4HB groups at 12 months. Histopathologic examination demonstrated mild to moderate neovascularization in all groups across time points.

To better understand the time course of macrophage phenotype transition from the pro‐inflammatory M1‐like to the tissue‐remodeling M2‐like within these neo‐nipples, M1 and M2 macrophage polarity was assessed as previously described.^14^ Abundant M1‐like macrophages (pro‐inflammatory) were seen throughout all 3D‐printed P4HB scaffolds at 1 month but decreased gradually over time, with few M1‐like macrophages noted after 12 months (Figure 7). Likewise, the infiltration of M2‐like macrophages (anti‐inflammatory) was observed in 3D‐printed P4HB scaffolds as early as 1 month after implantation, increasing between 3 and 6 months and predominating at later time points. In comparison, M2‐like macrophage infiltrate in mesh groups was greater and remained plentiful throughout the nipple constructs after 12 months. M1‐like macrophage expression in mesh groups was similar to that of the 3D‐printed P4HB scaffolds, which decreased gradually over time. The SIS group exhibited larger amounts of both M1‐like and M2‐like macrophage infiltrates at 1 month compared to the P4HB scaffolds but decreased sharply between 1 and 3 months as the implanted SIS material was rapidly degraded. Quantification of CD80 and CD206 expression verified the regenerative milieu (M1/M2 < 1) in all groups after 3 months, although qualitatively this ratio appeared greater for the P4HB devices (Figure 8a–g).

### 3.3 Kinetics of P4HB Scaffold degradation

#### 3.3.1 Scanning Electron Microscopy

SEM images were taken from the outer surface and cross‐sections of the scaffolds after the initial print and after 1, 3, 6, and 12 months in vivo (Figures 1, 5 and 6, Supplementary Figures [Link], [Link]). The smooth outer surface of continuous printed layers at pre‐implantation developed surface erosion after 1 month in vivo in the 3D‐printed P4HB scaffolds and was more apparent and widespread at all later time points. Less surface degradation of the interior latticework was noticed from the cross‐sectional view when compared to the outermost surface of the explanted 3D‐printed P4HB scaffolds. After 12 months, most P4HB filaments from the 3D‐sm, 3D‐25, and 3D‐30 groups were degraded, and there were no intact filament pieces salvageable after tissue digestion for SEM imaging (Figures [Link], [Link]). In contrast, minimal surface erosion was observed in mesh groups across all time points, both on the outermost surface and the interior mesh fibers, with only small fissures on the P4HB fibers visible by SEM, which is likely due to the fabrication process of the mesh filaments, which results in a compact linear orientation of P4HB within the fibers (Figure 6 and Figure S4).

#### 3.3.2 Molecular weight changes of explanted scaffolds

Analysis of the molecular weight of explanted P4HB scaffolds was also conducted to measure the kinetics of P4HB degradation in each group. The average molecular weight (Mw) of all 3D‐printed P4HB scaffolds decreased over time in vivo (3D‐sm: 304.2 kDa at 1 month and 44.8 kDa at 12 months; 3D‐20: 272.2 kDa at 1 month and 49.9 kDa at 12 months; 3D‐25: 315.7 kDa at 1 month and 46.2 kDa at 12 months: 3D‐30: 334.2 kDa at 1 month and 42.6 kDa at 12 months)^20^ (Figure 8i). The half‐life (T_1/2_) of 3D‐sm, 3D‐20, 3D‐25, and 3D‐30 groups after in vivo subcutaneous implantation was found to be 18, 22, 18, and 18 weeks, respectively. In contrast, the molecular weight of both mesh groups decreased to a lesser extent over 12 months (Mesh‐M: 303.1 kDa at 1 month and 65.4 kDa at 12 months; Mesh‐T: 271.2 kDa at 1 month and 61.8 kDa at 12 months) and the half‐life (T_1/2_) of both mesh groups was found to be 24 weeks after in vivo implantation. This is the same as the 24‐week half‐life (T_1/2_) of Mw for oriented mesh fibers from our previous study.^21^

#### 3.3.3 Biomechanical testing of explanted scaffolds

Manual compression of the neo‐nipples demonstrated signs of gradual softening of the reconstructed 3D‐printed P4HB nipples over time, especially between 6 and 12 months, at which point they were compressible with elastic rebound (Video S1, **3D‐20**; Video S2, **3D‐25**). Both manually rolled and mechanically thermoformed mesh groups showed good compressibility and elasticity, demonstrating compressive stiffness like native soft tissue both pre‐implantation as well as across all time points (Video S3, **Mesh‐T**). In contrast, by 6 months, the SIS group had no remaining resilience upon gentle compression due to the fast absorption of the implanted collagen matrix. Biomechanical testing showed an overall decrease in elastic modulus of the 3D‐printed P4HB scaffolds at 1 month, especially those groups with a higher stiffness at pre‐implantation (T0: 3D‐sm: 6.59 MPa, 3D‐20: 2.75 MPa, 3D‐25: 7.52 MPa, 3D‐30: 8.93 MPa), gradually approaching the previously reported human nipple reference stiffness (0.257 MPa) (At 12 months: 3D‐sm: 0.19 MPa, 3D‐20: 0.17 MPa, 3D‐25: 0.22 MPa, 3D‐30: 0.05 MPa)^22^ (Figure 8J). The stiffness of both mesh groups decreased slightly but remained essentially unchanged over 12 months within the range of 0.12 to 0.78 MPa. Similar to the loss of projection and volume, the SIS group demonstrated a dramatic decline in stiffness to only 0.0004 MPa after 12 months in vivo.

## 4 DISCUSSION

The creation of a nipple‐areola complex constitutes a vital, final step in the reconstruction of the breast following mastectomy. Although nipple reconstruction is a technically straightforward procedure, practically all described techniques suffer from a loss of long‐term projection, whether they are performed with autologous tissues alone or in combination with tissue substitutes (e.g., silicone, HA filler, etc.).^23^, ^24^, ^25^, ^26^, ^27^ Consequently, patient satisfaction with nipple reconstruction has been shown to be as low as 13%, primarily due to the significant loss of projection that consistently occurs postoperatively.^28^, ^29^ Although reconstructions utilizing biologic and synthetic materials are more efficacious in maintaining projection versus local skin flaps alone, these materials come with an increased risk of adverse complications (e.g., infection, extrusion, and unnatural firmness), stemming from a mismatch in biomaterial properties and local tissue requirements.^30^, ^31^ In previous work, we demonstrated the proof of concept of using 3D‐printed external P4HB scaffolds with or without the presence of an internal P4HB 3D latticework to engineer neo‐nipples with long‐term (1 year) projection. Although the scaffolds promoted neotissue formation in the approximate size and shape of a nipple, further optimization of the degradation rate of P4HB latticework was desired to ensure that the scaffold lost its initial unnatural stiffness while simultaneously generating a natural feeling nipple with consistent tissue ingrowth resulting in long‐term symmetric projection.

In the current study, we adjusted scaffold interior lattice infill density (20%, 25%, or 30%) and P4HB filament diameter (0.15 mm or 0.2 mm) without the presence of an external scaffold shell to determine the ideal latticework design that would best resist early skin contracture, promote tissue infiltration, and degrade at an appropriate rate to support a biocompatible local tissue response. Without an external shell, the surface area interface between the scaffold's three‐dimensional latticework and the host tissue promoted more rapid tissue infiltration. In addition, the flange along the base of the scaffold was added to enhance implant stability. Furthermore, to explore additional scaffold design elements, nipple scaffolds were also made from P4HB knitted mesh (available clinically as Phasix™ Mesh). These mesh scaffolds were either manually rolled and stabilized by fusing flat mesh to the base of the cylinder or thermoformed into a nipple shape with a bottom, rolled interior, and external shell fused together. Surgeons have anecdotally used custom‐made “rolled” P4HB mesh (off‐label) for years, but the practice has not gained widespread appeal, likely due to the inconsistent performance secondary to the non‐optimized intraoperative scaffold construction. All groups were compared to the only US FDA‐authorized device (Cook Biodesign® Nipple Cylinder) indicated for nipple reconstruction, although this device is made from porcine SIS composed primarily of collagen, rather than a macroporous scaffold constructed from an absorbable polyester. As a commonly used preclinical animal model for biomaterial studies, only male Sprague Dawley rats were used in this study to avoid the potentially confounding influence of estrogen.^32^ Four nipples per group per time point were created to gather sufficient data while keeping the overall number of animals used (58 rats) within a reasonable and manageable‐sized group (112 nipples). The statistical power of our findings may be limited due to the sample size; however, these preliminary results warrant larger follow‐on studies in a large animal model where more nipples can be implanted per animal to validate and expand upon the findings described herein.

All the nipple designs demonstrated a high biocompatibility with no complications/extrusion noticed over 12months of in vivo implantation. Compared to previous results from published studies in which reconstructed nipples flattened by at least 50% after 6 months and ~70 % after 1 year, our neo‐nipples reconstructed with 3D‐printed P4HB and mesh scaffolds maintained about 80% and 90%, respectively, of their projection after 12 months.^29^, ^33^, ^34^ In previous work, the 3D‐printed P4HB group encountered premature and often asymmetric buckling at the construct “waist” that caused a 25% loss of initial nipple projection as well as asymmetry in projection. We hypothesize that the external shell of those designs contributed to the buckling seen in the previous iteration since the shell contributed significantly to the peripheral construct support. Furthermore, the shell impeded rapid tissue invasion into the inner lattice. Removing the outer shell appeared to be the most important design change since none of the constructs in this study, including the group with the same filament diameter and infill design as the prior study, demonstrated asymmetric buckling. To our surprise, the 30% infill sustained a greater loss of projection and decrease in stiffness than the 20% and 25% infill groups (with the same diameter filaments), likely because the increased P4HB density induced an excessive inflammatory reaction and more rapid degradation. The slight loss of nipple projection in the 3D‐sm group can be attributed to the more rapid loss of integrity of the smaller diameter P4HB filaments between 3‐ and 6‐month post‐implantation, leading to a ~20% loss of neo‐nipple projection by 12 months. Most importantly, despite differences in initial stiffness between the P4HB constructs, after 12 months they all demonstrated stiffness approaching that of excised human nipple specimens.^13^, ^14^ Despite the current clinical utilization of acellular dermal matrix (ADM) and other decellularized collagen matrix scaffolds, such as the Cook Biodesign® Nipple Cylinder for nipple reconstruction, the SIS group encountered significant projection loss as early as 1 month and worsened over 1 year (only ~40% projection retained in the current study after 12 months), secondary to the rapid SIS degradation within 4 weeks after implantation.^35^, ^36^, ^37^ Even though rat skin is looser and thinner than human skin, it can still generate significant contractile forces as demonstrated by the considerable loss of projection in the SIS group, consistent with clinical observation.^37^

The mesh groups (especially Mesh‐T) showed promising results as an alternative P4HB design with consistently well‐preserved diameter and projection of neo‐nipples, increased amounts of fibrovascular tissue infiltration and collagen deposition in the scaffold, and favorable biomechanical properties over time. The P4HB fiber in the mesh groups undergoes a stretching and orientation process during its production that increases crystallinity and imparts strength. The oriented fibers are more resistant to enzymatic surface degradation than the 3D‐printed filaments, thus prolonging their strength and integrity in vivo, although the P4HB polymer loses molecular weight at similar rates by bulk hydrolysis independent of the method of manufacture. This led to the minimal degradation of both mesh groups over 12 months in our study. Manually rolling trimmed Phasix™ (P4HB) mesh strips to reconstruct nipples has been reported by individual plastic surgeons; however, the variability in the tightness of rolling and the number of mesh layers may affect the consistency of the outcomes of the reconstructed nipples, such as the projection and stiffness. As shown in the current study, we manually rolled 4–5 layers of mesh to form Mesh‐M nipple scaffolds with ~1 cm in diameter; while the overall projection preservation was above 80% over 12 months, it fluctuated between different time points, reflecting the variability of “handmade” devices. In contrast, the mechanically thermoformed mesh scaffold (Mesh‐T), in which the rolled mesh scaffold was covered by a molded bell‐shaped P4HB shell and thermally fused together, was manufactured to predefined sizes in a standard process that would provide a more consistent scaffold design for nipple reconstruction and reduce the preparation time in the operating room. Given the limited surface degradation of P4HB mesh fibers over 12 months, longer time observation of mesh groups is warranted to understand if their longevity predisposes them to extrusion or a maladaptive inflammatory response prior to complete degradation. Although no preclinical studies nor clinical cases to date have reported neoplasm formation associated with P4HB polymers (e.g., Phasix™ mesh), the implantation of solid foreign bodies (e.g., polyethylene, polyurethane, polymethylmethacrylate, silicone, and metals) into rodents has been associated with sarcoma formation in the surrounding tissues.^38^ Future work will test nipple reconstruction using 3D‐printed and mesh P4HB scaffolds in larger animal models (e.g., pigs) with skin and cutaneous healing more homologous to that of humans. This approach will provide more relevant insights for the clinical translation of this technology.

Taken together, these data suggest that 3D‐printed P4HB latticework scaffolds (20 ~ 25% infill density and 0.2 mm filament diameter) and mechanically thermoformed mesh scaffolds hold great potential as an off‐the‐shelf solution for nipple reconstruction, but longer in vivo observation time in larger animal models is warranted.

## 5 CONCLUSION

Engineered neo‐nipples formed from 3D‐printed P4HB and mechanically thermoformed mesh scaffolds demonstrated clinically meaningful diameter and projection maintenance over 12 months in a rat model. As the P4HB filaments degrade over time and the scaffold loses structural integrity, fibrovascular tissue in‐growth provides the structure that allows the engineered nipple to maintain proper shape, volume, and biomechanical properties approaching those of a native nipple.

## AUTHOR CONTRIBUTIONS

**Xue Dong:** Conceptualization; investigation; writing – original draft; methodology; validation; writing – review and editing; software; formal analysis; data curation. **Sophia Salingaros:** Investigation; writing – review and editing; writing – original draft. **Timothy Butler:** Conceptualization; methodology; validation; writing – review and editing; software; formal analysis; project administration; data curation; supervision; resources. **Skander Limem:** Conceptualization; writing – review and editing; project administration; supervision; resources. **Jason A. Spector:** Conceptualization; investigation; funding acquisition; writing – review and editing; methodology; project administration; supervision; resources.

## CONFLICT OF INTEREST STATEMENT

This research was in part supported by BD. Co‐authors Timothy Butler and Skander Limem are employed by BD. Timothy Butler completed the biomechanical testing. The BD team helped to review the data and final manuscript. The other authors declare no conflicts of interest.

There were no human subjects in this study, and informed consent was not applicable.

## Supporting information


**Figure S1.** Tissue assessment in 3D‐sm scaffolded neo‐nipples after in vivo implantation (left for peripheral, right for interior as indicated). H&E (first column) and Masson's Trichrome staining (second column) demonstrated overall cellular infiltration and collagen deposition in the nipple explants, accompanied with increased surface degradation of P4HB filaments overtime (seen in SEM images, third column). Few adipocytes were seen within scaffolds at 6 and 12 months. No intact filaments after tissue digestion were observed after 12 months. Scale bar = 100 μm.

**Figure S2.** Tissue assessment in 3D‐25 scaffolded neo‐nipples after in vivo implantation (left for peripheral, right for interior as indicated). H&E (first column) and Masson's Trichrome staining (second column) demonstrated overall cellular infiltration and collagen deposition in the nipple explants, accompanied with increased surface degradation of P4HB filaments overtime (seen in SEM images, third column). Less adipocytes were seen within scaffolds at 6 and 12 months. No intact filaments after tissue digestion were observed after 12 months. Scale bar = 100 μm.

**Figure S3.** Tissue assessment in 3D‐30 scaffolded neo‐nipples after in vivo implantation (left for peripheral, right for interior as indicated). H&E (first column) and Masson's Trichrome staining (second column) demonstrated overall cellular infiltration and collagen deposition in the nipple explants, accompanied with increased surface degradation of P4HB filaments overtime (seen in SEM images, third column). Few adipocytes were seen within scaffolds at 6 and 12 months. No intact filaments after tissue digestion were observed after 12 months. Scale bar = 100 μm.

**Figure S4.** Tissue assessment in Mesh‐M scaffolded neo‐nipples after in vivo implantation (left for peripheral, right for interior as indicated). H&E (first column) and Masson's Trichrome staining (second column) demonstrated overall cellular infiltration and collagen deposition in the nipple explants. Minimal surface degradation of mesh fibers was observed overtime (seen in SEM images, third column). Intact mesh fibers were gradually encapsulated by distinct layers of fibrous tissue at 6 and 12 months. Scale bar = 100 μm.

**Figure S5.** Tissue assessment in SIS scaffolded neo‐nipples after in vivo implantation (left for peripheral, right for interior as indicated). H&E (first column) and Masson's Trichrome staining (second column) demonstrated overall cellular infiltration and collagen distribution in the nipple explants. Decreased cell infiltrates were observed overtime. No identifiable scaffold was remaining for SEM imaging as early as 1 month post‐implantation. Scale bar = 100 μm.

**Video S1.** Manual compression of 3D‐20 scaffolded neo‐nipples at one year.

**Video S2.** Manual compression of 3D‐25 scaffolded neo‐nipples at one year.

**Video S3.** Manual compression of Mesh‐T scaffolded neo‐nipples at one year.

## Data Availability

The data that support the findings of this study are available from the corresponding author upon reasonable request.

## References

[1] American Cancer Society . Breast Cancer Facts & Figures 2022‐2024,Cancer Facts & Figures 2023. Available at: https://www.cancer.org/content/dam/cancer‐org/research/cancer‐facts‐and‐statistics/breast‐cancer‐facts‐and‐figures/2022‐2024‐breast‐cancer‐fact‐figures‐acs.pdf and https://www.cancer.org/content/dam/cancer‐org/research/cancer‐facts‐and‐statistics/annual‐cancer‐facts‐and‐figures/2023/2023‐cancer‐facts‐and‐figures.pdf

[2] Sibia US , Klune JR , Turcotte JJ , Holton LH 3rd , Riker AI . Hospital‐based same‐day compared to overnight‐stay mastectomy: an American College of Surgeons National Surgical Quality Improvement Program Analysis. Ochsner J. 2022;22(2):139‐145. 35756587 10.31486/toj.21.0103 PMC9196968

[3] Kim JH , Kang J , Najmiddinov B , Kim EK , Myung Y , Heo CY . Nipple projection change in immediate breast reconstruction and use of an acellular dermal matrix strut for maintaining nipple projection. Plast Reconstr Surg. 2023;152(5):949‐957. 36877621 10.1097/PRS.0000000000010355

[4] Perez‐Otero S , Hemal K , Boyd CJ , et al. Minimizing nipple‐areolar complex complications in Prepectoral breast reconstruction after nipple‐sparing mastectomy. Ann Plast Surg. 2024;92(4S Suppl 2):S179‐S184. 38556670 10.1097/SAP.0000000000003906

[5] Guyomard V , Leinster S , Wilkinson M . Systematic review of studies of patients' satisfaction with breast reconstruction after mastectomy. Breast. 2007;16(6):547‐567. 18024116 10.1016/j.breast.2007.04.004

[6] Wellisch DK , Schain WS , Noone RB , Little JW 3rd . The psychological contribution of nipple addition in breast reconstruction. Plast Reconstr Surg. 1987;80(5):699‐704. 3671562 10.1097/00006534-198711000-00007

[7] Little JW 3rd . Nipple‐areola reconstruction. Clin Plast Surg. 1984;11(2):351‐364. 6373095

[8] Momoh AO , Colakoglu S , de Blacam C , et al. The impact of nipple reconstruction on patient satisfaction in breast reconstruction. Ann Plast Surg. 2012;69(4):389‐393. 22868326 10.1097/SAP.0b013e318246e572

[9] Maistriaux L , Foulon V , Fievé L , et al. Reconstruction of the human nipple‐areolar complex: a tissue engineering approach. Front Bioeng Biotechnol. 2024;11:1295075. 38425730 10.3389/fbioe.2023.1295075 PMC10902434

[10] Haslik W , Nedomansky J , Hacker S , Nickl S , Schroegendorfer KF . Objective and subjective evaluation of donor‐site morbidity after nipple sharing for nipple areola reconstruction. J Plast Reconstr Aesthet Surg. 2015;68(2):168‐174. 25465146 10.1016/j.bjps.2014.10.023

[11] Paolini G , Firmani G , Briganti F , Sorotos M , di Santanelli Pompeo F . Guiding nipple‐areola complex reconstruction: literature review and proposal of a new decision‐making algorithm. Aesth Plast Surg. 2021;45(3):933‐945. 10.1007/s00266-020-02047-9 PMC8144123 33216178

[12] Cazzato V , Stocco C , Scian A , et al. Nipple reconstruction using the “arrow flap” technique: outcomes and patients satisfaction. Clin Breast Cancer. 2024;24(4):e226‐e231. doi:10.1016/j.clbc.2024.01.011 38503614

[13] Dong X , Shih S , Premaratne ID , et al. Long‐term maintenance of projection of nipples reconstructed using three‐dimensionally printed Poly‐4‐hydroxybutyrate bioabsorbable scaffolds. Plast Reconstr Surg. 2023;152(4):646e‐654e. doi:10.1097/prs.0000000000010384 36877752

[14] Dong X , Premaratne ID , Sariibrahimoglu K , et al. 3D‐printed poly‐4‐hydroxybutyrate bioabsorbable scaffolds for nipple reconstruction. Acta Biomater. 2022;143:333‐343. 35240316 10.1016/j.actbio.2022.02.040

[15] Guerid S , Boucher F , Mojallal A . Nipple reconstruction using rib cartilage strut in microsurgical reconstructed breast. Ann Chir Plast Esthet. 2017;62(4):332‐335. 28262373 10.1016/j.anplas.2017.02.002

[16] Utsunomia C , Ren Q , Zinn M . Poly(4‐hydroxybutyrate): current state and perspectives. Front Bioeng Biotechnol. 2020;8:257. 32318554 10.3389/fbioe.2020.00257 PMC7147479

[17] Winocour S , Saksena A , Oh C , et al. A systematic review of comparison of autologous, allogeneic, and synthetic augmentation grafts in nipple reconstruction. Plast Reconstr Surg. 2016;137(1):14e‐23e. doi:10.1097/prs.0000000000001861 26710046

[18] Jendrossek D . Microbial degradation of polyesters. Adv Biochem Eng Biotechnol. 2001;71:293‐325. 11217416 10.1007/3-540-40021-4_10

[19] Tarazona NA , Machatschek R , Lendlein A . Influence of depolymerases and lipases on the degradation of Polyhydroxyalkanoates determined in Langmuir degradation studies. Adv Mater Interfaces. 2020;7:2000872.

[20] Williams SF , Rizk S , Martin DP . Poly‐4‐hydroxybutyrate (P4HB): a new generation of resorbable medical devices for tissue repair and regeneration. Biomed Tech (Berl). 2013;58(5):439‐452. 23979121 10.1515/bmt-2013-0009

[21] Martin DP , Williams SF . Medical applications of poly‐4‐hydroxybutyrate: a strong flexible absorbable biomaterial. Biochem Eng J. 2003;16(2):97‐105.

[22] Samadi A , Premaratne ID , Wright MA , et al. Nipple engineering: maintaining nipple geometry with externally scaffolded processed autologous costal cartilage. J Plast Reconstr Aesthet Surg. 2021;74(10):2596‐2603. 33863678 10.1016/j.bjps.2021.03.010

[23] Khoo D , Ung O , Blomberger D , Hutmacher DW . Nipple reconstruction: a regenerative medicine approach using 3D‐printed tissue scaffolds. Tissue Eng Part B Rev. 2019;25(2):126‐134. 30379123 10.1089/ten.TEB.2018.0253

[24] Gougoutas AJ , Said HK , Um G , Chapin A , Mathes DW . Nipple‐Areola Complex Reconstruction. Plast Reconstr Surg. 2018;141(3):404e‐416e. 10.1097/PRS.0000000000004166 29481412

[25] Craft RO , May JW Jr . Staged nipple reconstruction with vascularized SurgiMend acellular dermal matrix. Plast Reconstr Surg. 2011;127(6):148e‐149e. doi:10.1097/prs.0b013e3182131e74 21617431

[26] Panettiere P , Marchetti L , Accorsi D . Filler injection enhances the projection of the reconstructed nipple: an original easy technique. Aesth Plast Surg. 2005;29(4):287‐294. 10.1007/s00266-004-0145-y 16044237

[27] Jankau J , Jaśkiewicz J , Ankiewicz A . A new method for using a silicone rod for permanent nipple projection after breast reconstruction procedures. Breast. 2011;20(2):124‐128. 21115347 10.1016/j.breast.2010.10.001

[28] Jabor MA , Shayani P , Collins DR Jr , Karas T , Cohen BE . Nipple‐areola reconstruction: satisfaction and clinical determinants. Plast Reconstr Surg. 2002;110(2):457‐463. 12142660 10.1097/00006534-200208000-00013

[29] Shestak KC , Gabriel A , Landecker A , Peters S , Shestak A , Kim J . Assessment of long‐term nipple projection: a comparison of three techniques. Plast Reconstr Surg. 2002;110(3):780‐786. 12172139 10.1097/00006534-200209010-00010

[30] Satteson ES , Reynolds MF , Bond AM , Pestana IA . An analysis of complication risk factors in 641 nipple reconstructions. Breast J. 2016;22(4):379‐383. 27038175 10.1111/tbj.12591

[31] Momeni A , Ghaly M , Gupta D , et al. Nipple reconstruction: risk factors and complications after 189 procedures. Eur J Plast Surg. 2013;36(10):633‐638. 24072956 10.1007/s00238-013-0841-4 PMC3780439

[32] Hatt LP , Thompson K , Helms JA , Stoddart MJ , Armiento AR . Clinically relevant preclinical animal models for testing novel cranio‐maxillofacial bone 3D‐printed biomaterials. Clin Transl Med. 2022;12(2):e690. 35170248 10.1002/ctm2.690 PMC8847734

[33] Nahabedian MY . Secondary nipple reconstruction using local flaps and AlloDerm. Plast Reconstr Surg. 2005;115(7):2056‐2061. 15923855 10.1097/01.prs.0000164490.99581.f9

[34] Few JW , Marcus JR , Casas LA , Aitken ME , Redding J . Long‐term predictable nipple projection following reconstruction. Plast Reconstr Surg. 1999;104(5):1321‐1324. 10513912 10.1097/00006534-199910000-00012

[35] Konstantinovic ML , Lagae P , Zheng F , Verbeken EK , de Ridder D , Deprest JA . Comparison of host response to polypropylene and non‐cross‐linked porcine small intestine serosal‐derived collagen implants in a rat model. BJOG. 2005;112(11):1554‐1560. 16225578 10.1111/j.1471-0528.2005.00688.x

[36] Bramhall RJ , Thiruchelvam PTR , Concepcion M , Gui GP . Use of acellular dermal matrix (ADM) in nipple reconstruction: the ‘central‐pillar technique’. Gland Surg. 2017;6(4):394‐398. 28861381 10.21037/gs.2017.03.13 PMC5566655

[37] Collins B , Williams JZ , Karu H , Hodde JP , Martin VA , Gurtner GC . Nipple reconstruction with the biodesign nipple reconstruction cylinder: a prospective clinical study. Plast Reconstr Surg Glob Open. 2016;4(8):e832. 27622100 10.1097/GOX.0000000000000846 PMC5010323

[38] Greaves P , Chouinard L , Ernst H , et al. Proliferative and non‐proliferative lesions of the rat and mouse soft tissue, skeletal muscle and mesothelium. J Toxicol Pathol. 2013;26(3 Suppl):1S‐26S. 25035576 10.1293/tox.26.1S PMC4091527

