# Supplementary figures and images for: Optimizing design parameters of 3D‐printed poly‐4‐hydroxybutyrate nipple scaffolds for nipple reconstruction
Source: Bioeng Transl Med. 2025 Apr 7;10(4):e70010. doi: 10.1002/btm2.70010 (PMC12284436; doi:10.1002/btm2.70010)

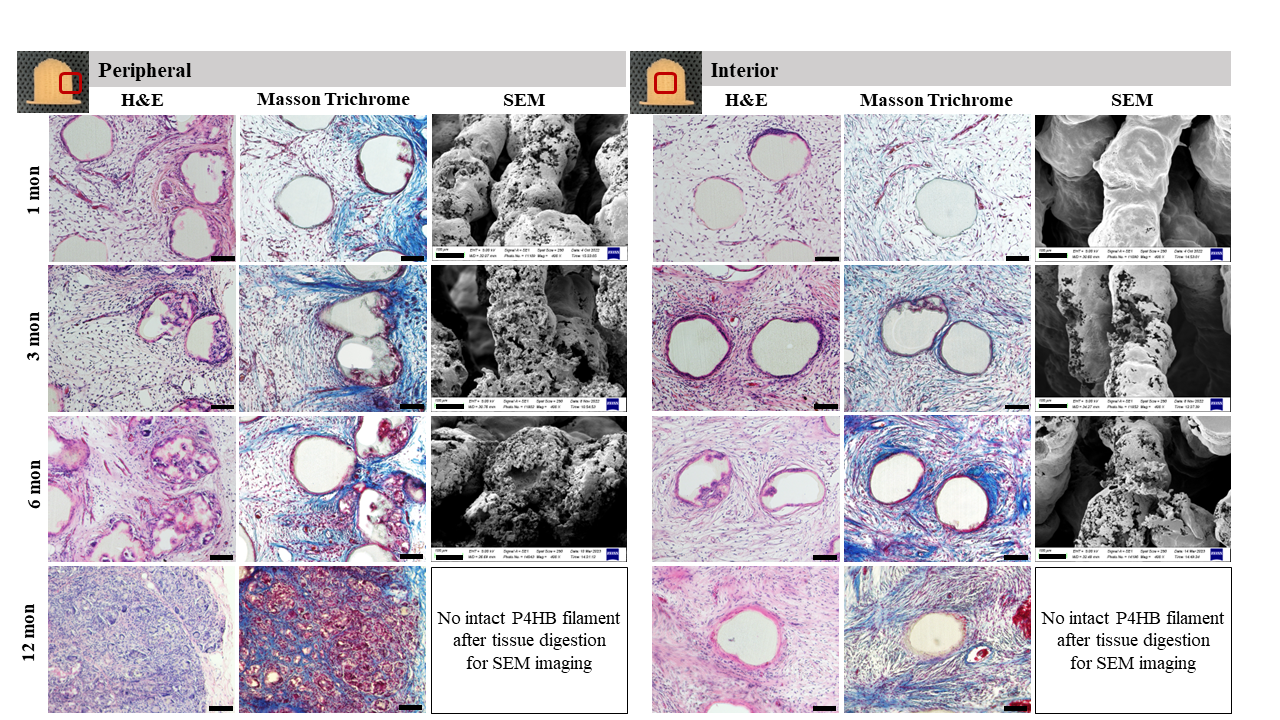

Supplement: Supplementary file 1 — Figure S1. Tissue assessment in 3D‐sm scaffolded neo‐nipples after in vivo implantation (left for peripheral, right for interior as indicated). H&E (first column) and Masson's Trichrome staining (second column) demonstrated overall cellular infiltration and collagen deposition in the nipple explants, accompanied with increased surface degradation of P4HB filaments overtime (seen in SEM images, third column). Few adipocytes were seen within scaffolds at 6 and 12 months. No intact filaments after tissue digestion were observed after 12 months. Scale bar = 100 μm. [file BTM2-10-e70010-s006.tif]

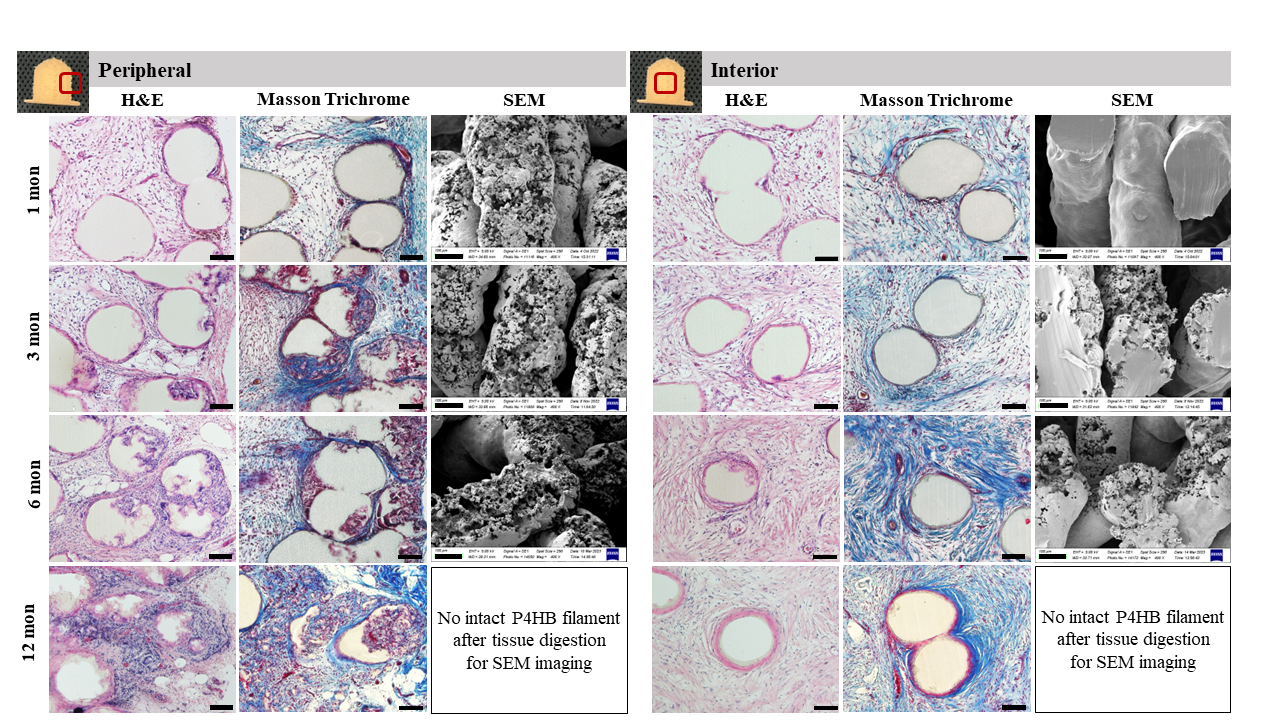

Supplement: Supplementary file 2 — Figure S2. Tissue assessment in 3D‐25 scaffolded neo‐nipples after in vivo implantation (left for peripheral, right for interior as indicated). H&E (first column) and Masson's Trichrome staining (second column) demonstrated overall cellular infiltration and collagen deposition in the nipple explants, accompanied with increased surface degradation of P4HB filaments overtime (seen in SEM images, third column). Less adipocytes were seen within scaffolds at 6 and 12 months. No intact filaments after tissue digestion were observed after 12 months. Scale bar = 100 μm. [file BTM2-10-e70010-s002.tif]

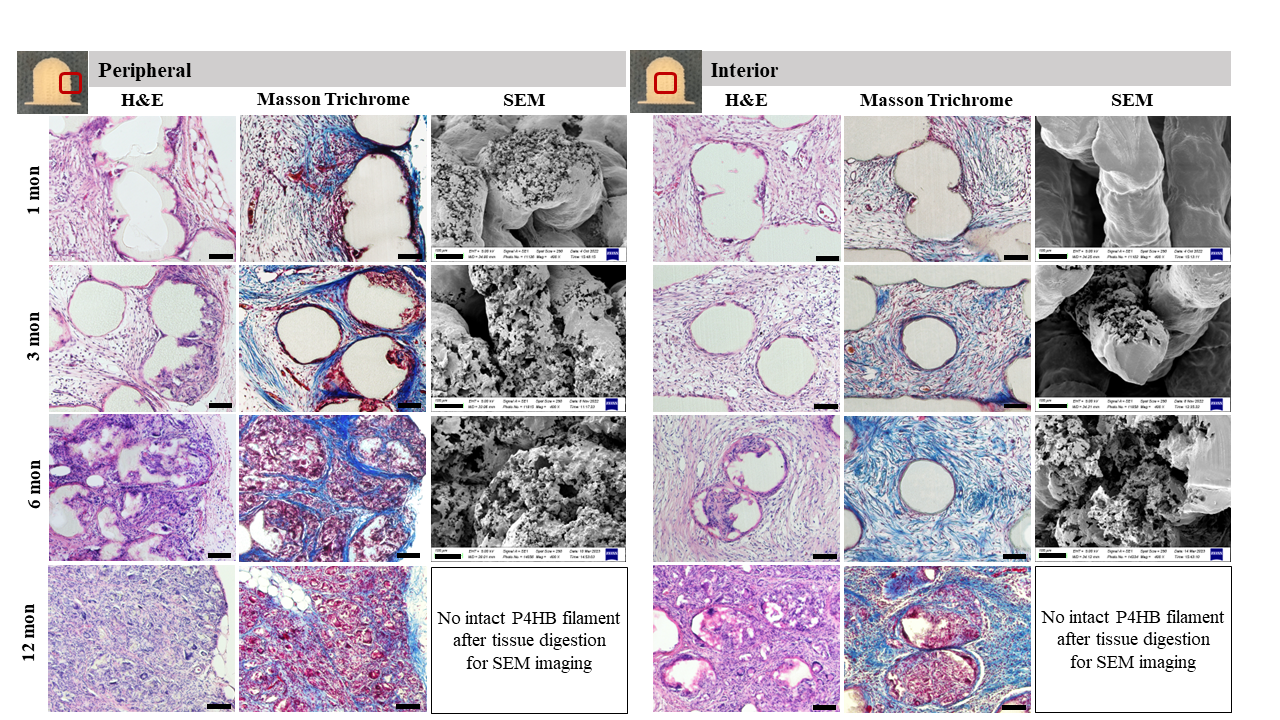

Supplement: Supplementary file 3 — Figure S3. Tissue assessment in 3D‐30 scaffolded neo‐nipples after in vivo implantation (left for peripheral, right for interior as indicated). H&E (first column) and Masson's Trichrome staining (second column) demonstrated overall cellular infiltration and collagen deposition in the nipple explants, accompanied with increased surface degradation of P4HB filaments overtime (seen in SEM images, third column). Few adipocytes were seen within scaffolds at 6 and 12 months. No intact filaments after tissue digestion were observed after 12 months. Scale bar = 100 μm. [file BTM2-10-e70010-s007.tif]

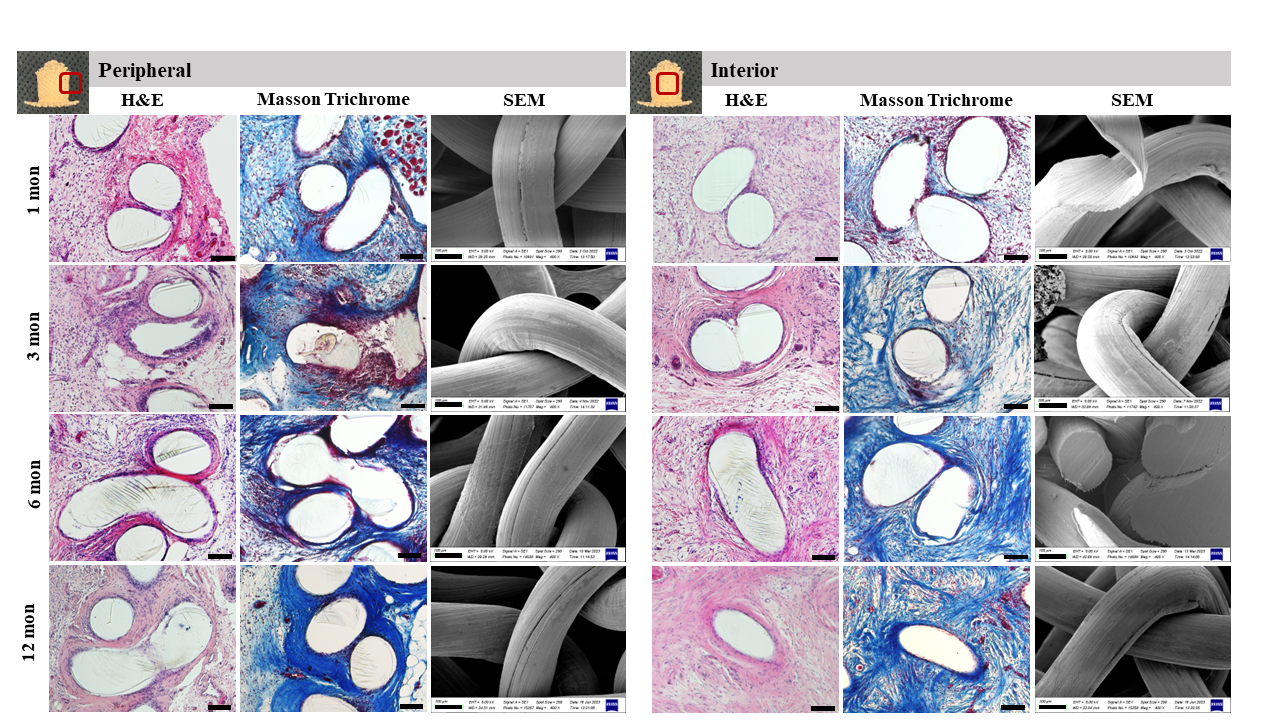

Supplement: Supplementary file 4 — Figure S4. Tissue assessment in Mesh‐M scaffolded neo‐nipples after in vivo implantation (left for peripheral, right for interior as indicated). H&E (first column) and Masson's Trichrome staining (second column) demonstrated overall cellular infiltration and collagen deposition in the nipple explants. Minimal surface degradation of mesh fibers was observed overtime (seen in SEM images, third column). Intact mesh fibers were gradually encapsulated by distinct layers of fibrous tissue at 6 and 12 months. Scale bar = 100 μm. [file BTM2-10-e70010-s001.tif]

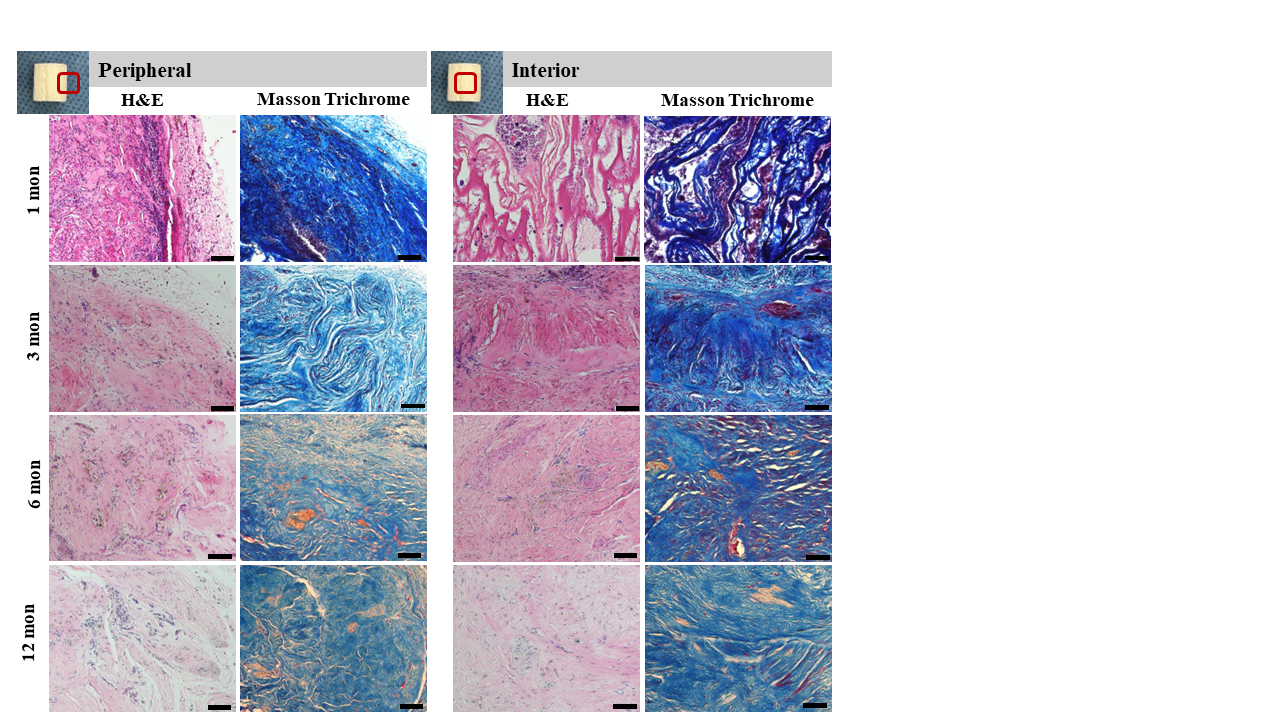

Supplement: Supplementary file 5 — Figure S5. Tissue assessment in SIS scaffolded neo‐nipples after in vivo implantation (left for peripheral, right for interior as indicated). H&E (first column) and Masson's Trichrome staining (second column) demonstrated overall cellular infiltration and collagen distribution in the nipple explants. Decreased cell infiltrates were observed overtime. No identifiable scaffold was remaining for SEM imaging as early as 1 month post‐implantation. Scale bar = 100 μm. [file BTM2-10-e70010-s003.tif]
